# Therapeutic potential of the phosphino Cu(I) complex (HydroCuP) in the treatment of solid tumors

**DOI:** 10.1038/s41598-017-13698-1

**Published:** 2017-10-24

**Authors:** Valentina Gandin, Cecilia Ceresa, Giovanni Esposito, Stefano Indraccolo, Marina Porchia, Francesco Tisato, Carlo Santini, Maura Pellei, Cristina Marzano

**Affiliations:** 1 Dipartimento di Scienze del Farmaco, Università degli Studi di Padova, via Marzolo 5, 35131 Padova, Italy; 2 Experimental Neurology Unit and Milan Center for Neuroscience, School of Medicine and Surgery, University of Milano-Bicocca, Via Cadore 48, 20900 Monza, MB Italy; 3 Istituto Oncologico Veneto IOV - IRCCS, 35128 Padova, Italy; 4 CNR-ICMATE, Corso Stati Uniti 4, 35127 Padova, Italy; 5 School of Science and Technology – Chemistry Division, University of Camerino, via S. Agostino 1, 62032 Camerino, MC Italy

## Abstract

[Cu(thp)_4_][PF_6_] (HydroCuP) is a phosphino copper(I) complex highly soluble and stable in physiological media that has been developed as a possible viable alternative to platinum-based drugs for anticancer therapy. HydroCuP potently inhibited the growth of human cancer cells derived from solid tumors by inducing endoplasmatic reticulum (ER) stress thus leading to cell death through paraptosis with a preferential efficacy against cancer rather than non-cancer cells. Aim of the present study was to assess the therapeutic potential of HydroCuP *in vivo*, in syngenic and xenograft murine models of solid tumors by triggering the Unfolded Protein Response (UPR) pathway. With respect to platinum drugs, HydroCuP induced a markedly higher reduction of tumor growth associated with minimal animal toxicity. In human colorectal cancer xenografts, chemotherapy with HydroCuP was extremely effective in both oxaliplatin-sensitive and resistant models. The favorable *in vivo* tolerability of HydroCuP was also correlated to an encouraging biodistribution profile. Additionally, no signs of drug-related neurotoxicity and nephrotoxicity were observed. Altogether, these results demonstrate that HydroCuP appears worth of further investigation to evaluate its therapeutic activity towards a broad spectrum of solid malignancies.

## Introduction

Although highly effective toward a number of solid tumors^1^ platinum anticancer drugs (cisplatin, CDDP, and the second and third generation analogues carboplatin and oxaliplatin, OXP)^2^ cause severe toxic effects on normal tissues and induce early appearance of resistance phenomena, even at the beginning of their administration or after the first therapeutic cycles^1,3,4^. These drawbacks have stimulated an extensive search to develop alternative strategies based on different metallodrugs with improved pharmacological properties and aimed at different targets^5,6^. Many coordination compounds that can attack cancer interfering with biological processes other than DNA replication have been proposed^7^. Examples of alternative molecular targets for metal-based drugs include thiol-containing proteins, proteasome, matrix metalloproteases, telomerases, topoisomerases, glutathione-S-transferases, and histone deacetylases^8^.

DNA damaging agents, like platinum drugs, promote DNA lesions that stall DNA replication and collapse replication forks, resulting in cell death. However, if not repaired properly, many of these genomic insults can induce gene mutations or chromosomal alterations. It is noteworthy that some patients treated with cisplatin develop cancer due to cisplatin-induced DNA lesions approximately 10 years after therapy^8^. Thus, developing anticancer agents that kill rapidly dividing cells with minimal potentially deadly side effects of chromosomal alterations and mutagenesis would be highly desirable.

In this field, copper complexes showed encouraging perspectives^9–13^. Copper-based complexes have been investigated on the assumption that endogenous metals may be less toxic for normal cells with respect to cancer cells. The altered metabolism of cancer cells and differential response between normal and tumor cells to copper are the basis for the development of copper complexes endowed with antineoplastic characteristics.

Recent findings have confirmed that copper complexes represent good alternatives to platinum drugs^14^. Actually, copper species, besides possessing a broader spectrum of activity and a lower toxicity, are able to overcome inherited and/or acquired resistance to cisplatin. These features are consistent with the hypothesis that copper complexes possess mechanism(s) of action different from those shown by platinum drugs. So far, little information is available on the molecular basis for the mode of action of copper complexes. At present, most investigations still focus on the potential ability of these complexes or fragments thereof, to interact with DNA. However, other cellular constituents such as topoisomerases or the proteasome multiprotein complex are emerging as new putative targets^14–17^.

Since Cu(I) is the chemical form generally accepted by the bioinorganic community to describe the active internalization of physiological copper in mammalian cells through copper transporter (CTR) proteins, examples of Cu(I) complexes displaying antitumor potential are being developed day by day^14^.

However, for very few of them the *in vivo* activity has been evaluated. This is likely related to the intrinsic difficulty to stabilize copper(I) species, especially in aqueous media. Thus tuning the hydrolysis and the activation of the redox machinery to minimize off-target binding in blood while maintaining enough reactivity to inhibit the cellular target, should be a guiding principle in the design of novel anticancer copper(I) complexes.

Hydrophilic tertiary phosphanes (P) have been used to obtain stable, water-soluble [Cu(P)_4_]^+^-type species that proved to be easy to handle during *in vitro* tests and showed promising antiproliferative effects^18,19^. Among them, the monocationic [Cu(thp)_4_][PF_6_] complex (HydroCuP) (thp = tris-hydroxymethylphosphine) showed an excellent *in vitro* antitumor activity against a wide range of solid tumors, including platinum drug refractory/resistant tumors^20^. Moreover, HydroCuP was much less cytotoxic against non-tumor cells than Pt(II) drugs with selectivity index (SI, the quotient of the average IC_50_ toward non-malignant cells divided by the average IC_50_ for the malignant cells) values about 35- and 10-fold higher than those calculated for CDDP and OXP, respectively^21^. Electrospray ionization mass spectrometry (ESI-MS) studies revealed that the original [Cu(P)_4_]^+^ pro-drugs underwent dissociation with production of coordinative unsaturated [Cu(P)_3_]^+^ and [Cu(P)_2_]^+^ species, which represented key intermediates for the activation of potential biological properties^22^. Furthermore, by means of a mechanistic detailed study we proposed the involvement of human copper transporter 1, hCtr1, in the mechanisms for HydroCuP cellular entry^23^. Although expression of hCtr1 is ubiquitous because all the tissues require copper, studies showed that expression levels were highly variable among normal tissues and also human malignancies^14^.

By molecular and cellular studies, HydroCuP was found to inhibit chymotrypsin-like (CT-L), trypsin-like (T-L) and caspase-like (C-L) catalytic activities of 26S proteasome causing intracellular accumulation of polyubiquitinated proteins and functional suppression of the ubiquitin-proteasome pathway, thus triggering endoplasmic reticulum (ER) stress and the concomitant induction of unfolded protein response (UPR). The irreversible ER stress was accompanied with a massive cytoplasmatic vacuolization and triggering of a type of a programmed cell death (PCD) alternative to apoptosis and termed paraptosis^21^. The ability to activate paraptosis makes HydroCuP a very promising agent to tackle apoptosis resistance in cancer cells. It is worth mentioning that HydroCuP was found particularly effective against a series of human cultured colon cancer cells^21^. Colorectal cancer is at the top of the list of the most common cancers worldwide, with around 1 million new cases diagnosed every year^24^. Early-stage colorectal cancer is frequently curable with surgery, but the appearance of metastases often leads to fatal consequences for the patient^25^. Oxaliplatin is a third generation platinum compound and the first platinum-based compound to show efficacy in the treatment of colorectal cancer^26^ and approved for therapy as a front-line agent^27^. It is a key drug in FOLFOX (folinic acid, 5-fluorouracil, and OXP) regimen for the treatment of colorectal cancer. Similarly to CDDP, the efficacy of OXP is limited by the development of cellular resistance. Moreover, it causes severe and disabling sensory peripheral neurotoxicity due to accumulation of the drug in the dorsal root ganglia (DRG), where sensory neurons are located^28,29^. Presently, there are no other drugs in advanced clinical development for the treatment of patients with oxaliplatin-refractory colorectal cancer.

Based on these premises, in this study we performed a pre-clinical investigation on the therapeutic potential of HydroCuP in 3D colon cancer cell cultures and in three independent murine models, including the highly aggressive Lewis Lung Carcinoma (LLC) as an example of syngeneic murine model and two mouse xenograft models, the LoVo colorectal oxaliplatin-sensitive xenograft and the LoVo colorectal oxaliplatin-resistant xenograft.

The novel phosphino copper(I) complex, HydroCuP, suppressed tumor growth in both the syngeneic and xenograft models, being also able to overcome OXP-resistance without causing evident toxic or side effects. To our knowledge, this is the first copper(I) complex reported to inhibit cell growth of oxaliplatin-resistant cancer cells in an *in vivo* tumor model.

## Results and Discussion

### *In vitro* antitumor activity on 3D colon cancer cell cultures

The effect of HydroCuP on different panels of 2D human cancer cell cultures had been already evaluated^19–21^, showing that HydroCuP yielded IC_50_ values ranging from less than 0.3 µM to 3 µM being up to 50- and 30-fold more cytotoxic than CDDP and OXP, respectively. Notably, HydroCuP showed a significant antiproliferative activity against cancer cells derived from solid tumours, including mainly colon carcinoma cells. However, it has been recently well described that in two-dimensional (2D) monolayer cell cultures cellular activities are often altered and their typical *in vivo* functions resulted to be lost. As a result, the conventional 2D cell culture provides limited predictive capacity for drug testing.

As attractive alternative, 3D cell cultures have proven to be a physiologic mimic of the *in vivo* tissue because they produce a similar cellular microenvironment^30^. Actually, on a three-dimensional architectural organization, tumor cells are not uniformly exposed to nutrients or oxygen *in vitro*, thus closely mimicking the organization of human tumors. Furthermore, significant differences were described in terms of sensitivity to drugs among 2D and 3D cultures due to significant differences in terms of growth, migration, morphology and gene expression^30^.

**Figure 1:**
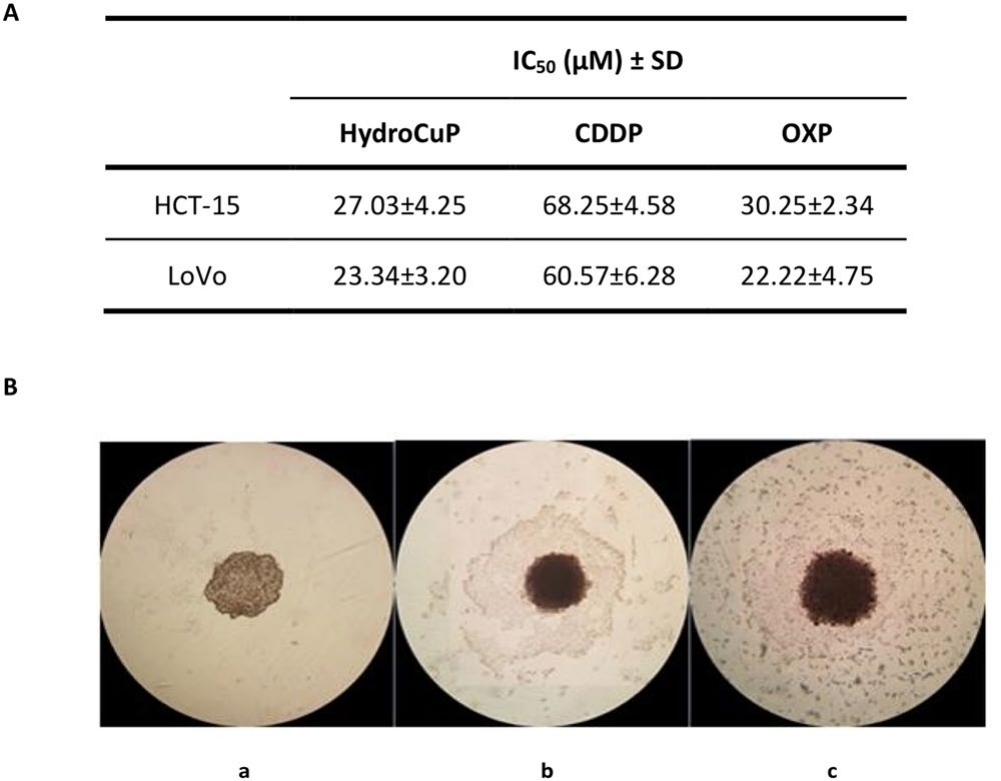
Activity in 3D cell cultures. (**A**) Spheroids (2.5 × 10^3^ cells/well) were treated for 72 h with increasing concentrations of tested compounds. The growth inhibitory effect was evaluated by means of APH test. IC_50_ values were calculated from the dose-survival curves by four parameter logistic model (P < 0.05). SD = standard deviation. (**B**) Representative images (10x) of HCT-15 3D culture: a = controls; b = spheroids treated with IC_50_ concentrations of HydroCuP for 24 h; c = spheroids treated with IC_50_ concentrations of HydroCuP for 48 h.

In order to improve the predictive capacity of the *in vitro* assays, we tested HydroCuP activity in two 3D cell cultures of human colon cancer cells, HCT-15 and LoVo. The colon cancer spheroids were treated with HydroCuP for 72 h and the cell viability was assessed by means of the acid phosphatase (APH) assay (Fig. 1, panel A). For comparison purposes, the cytotoxicities of CDDP and OXP were evaluated in the same experimental conditions.

In both colon cancer spheroid models, HydroCuP yielded IC_50_ values very similar to those of OXP and about 2.5 lower than those obtained with CDDP. In addition, monitoring the morphology of the spheroids (see Fig. 1, panel B), whereas untreated spheroids showed a compact and cohesive morphology (a), HydroCuP-treatment induced a time-dependent disaggregation of spheroids, determining a substantial decrease in the spheroid compactness (b and c).

### *In vivo* antitumor activity towards Lewis Lung Carcinoma (LLC)

The *in vivo* antitumor activity of HydroCuP was firstly tested in the highly aggressive, poorly immunogenic mouse LLC implanted intramuscularly (i.m.) in C57BL mice. The tumour growth inhibition induced by treatment with copper(I) complex was evaluated both in LLC model and compared with that promoted by the reference metallo-drug, CDDP.

The schedule of HydroCuP administration *in vivo* was initially determined by assessing the total dose that could be injected without undue toxicity for different schedules in nontumor-bearing C57BL mice (data not shown). HydroCuP has been administered in aqueous solution taking advantage of its excellent water solubility (>2.0 mg/mL at pH 7.4) and good solution stability, enabling *in vivo* formulation using 0.9% NaCl normal saline^21,22^. Three schedules of i.p. HydroCuP were administered: early treatment (days 3, 5, 7 and 9 after tumor inoculum), intermediate treatment (days 7–14) and late treatment with split-doses (days 9–11 with a loading dose and days 12–14 with a lower maintenance dose). Table 1 shows the results obtained in LLC-bearing mice following the different treatment schedules. After 24 h from tumor implantation, mice were randomly divided into five groups (8 animals per group, 10 controls). Cisplatin treatment schedule was selected according to standard protocols designed to optimize its efficacy and minimize the occurrence of adverse events^31^. For the early treatment, control mice received the vehicle (0.9% NaCl). HydroCuP was dosed at 25, 35 and 50 mg/kg i.p. on days 3, 5, 7, 9, 11 and 13 after tumor implantation. Cisplatin was dosed at 1.5 mg/kg i.p. on days 3, 5, 7, 9, 11 and 13 after tumor implantation. At day 15, control and treated animals were sacrificed, and the inhibition of tumor growth was evaluated.

**Table 1:** Treatment of LLC.

Compound	Dose (mg·kg^−1^)	Average tumor weight (mean ± S.D., g)	Inhibition of tumor growth (%)
**Early treatment**			
control^a^	—	0.638 ± 0.01	—
HydroCuP	25	0.473 ± 0.12*	25.86
HydroCuP	35	0.273 ± 0.04**	57.21
HydroCuP	50	0.113 ± 0.04**	82.28
CDDP	1.5	0.168 ± 0.10**	73.66
**Intermediate treatment**			
control^a^	—	0.502 ± 0.16	—
HydroCuP	30	0.088 ± 0.03**	82.37
HydroCuP	50	0.071 ± 0.02**	85.85
CDDP	1.5	0.061 ± 0.03**	87.84
**Late treatment**			
control^a^	—	0.432 ± 0.21	—
HydroCuP	50 (days 9–11) 30 (days 12–14)	0.024 ± 0.03**	94.44
CDDP	1.5	0.118 ± 0.10**	72.68

^a^vehicle (0.9% NaCl).

Lewis lung carcinoma (LLC) was implanted i.m. into the right hind leg of 8-week old imbred C57BL mice. After 24 h from tumor implantation, mice were randomly divided into groups of 8 animals (10 controls).

Early treatment: HydroCuP was dosed at 25, 35 and 50 mg/kg i.p. on days 3, 5, 7, 9, 11 and 13 after tumor implantation. CDDP was dosed at 1.5 mg/kg i.p. on days 3, 5, 7, 9, 11 and 13 after tumor implantation.

Intermediate treatment: Chemotherapy was delayed until the tumor became visible (day 7). Day 7–14: animal received 30 and 50 mg/kg of HydroCuP or 1.5 cisplatin mg/kg daily i.p.

Late treatment: Chemotherapy was delayed until the tumor became palpable (day 9). From day 9 to day 11, HydroCuP was dosed daily at 50 mk/kg i.p. whereas from day 12 to day 14 at 30 mg/kg i.p. CDDP was dosed daily at 1.5 mg/kg i.p.

At day 15 animals were sacrificed, legs amputated at the proximal end of the femur, and the inhibition of tumor growth was determined as the difference in weight of the tumor-bearing leg and the healthy leg expressed as percentage referred to the control animals. *p < 0.05, **p < 0.01.

**Figure 2:**
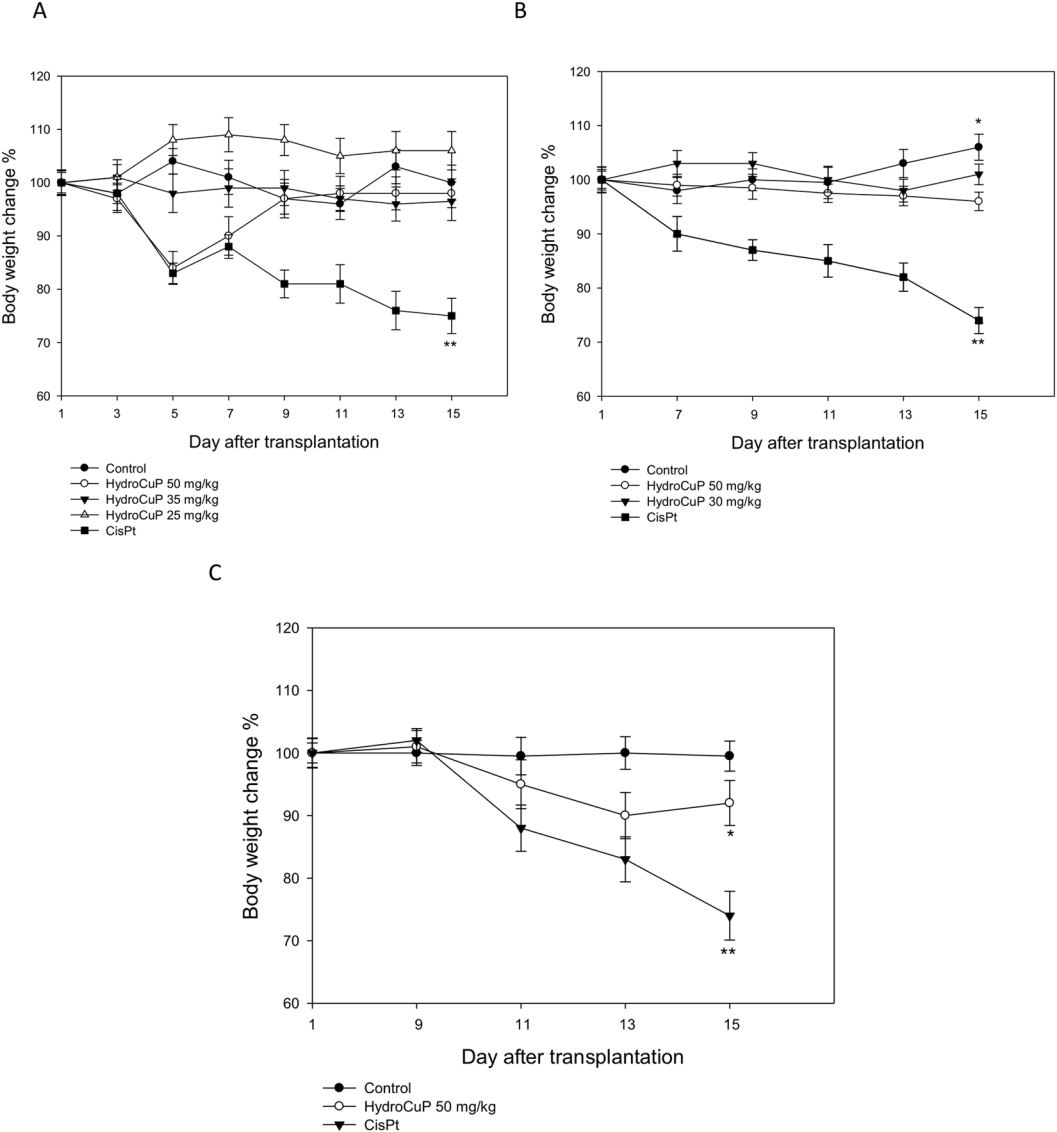
Body weight changes. The body weight changes of LLC bearing-C57BL mice treated with vehicle or tested compounds following early (**A**), intermediate (**B**) and late (**C**) treatments. Weights were measured every two days. Error bars indicate the S.D. **P < 0.01; *P < 0.05.

As shown in Table 1, HydroCuP treatment resulted in a dose-dependent inhibition of proliferation of tumor cell population. HydroCuP exerted a statistically significant antitumor activity compared to vehicle-treated mice (P < 0.05), even at the lower daily dose of 25 mg/kg with a tumor growth inhibition of 26%. Mice treated with 50 mg/kg of HydroCuP showed a tumor growth inhibition slightly higher to that observed for mice treated with 1.5 mg/kg of cisplatin. Over the course of 15 days, changes in the body weight of tumor-bearing mice were daily monitored (Fig. 2, panel A). Chemotherapy with HydroCuP did not induce significant body weight loss and no signs of discomfort were evident, whereas mice treated with cisplatin appeared prostrate and showed substantial weight loss.

The most rigorous preclinical evaluation of an antineoplastic agent is to determine its ability to induce responses in well-established tumors. To test the therapeutic efficacy of HydroCuP in animals with advanced disease, LLC tumors were allowed to establish and grow to visible and palpable size before the start of chemotherapy. In the intermediate treatment schedules, from day seven after tumor inoculation (visible tumor) LLC-bearing mice were daily treated i.p. with HydroCuP at 30 and 50 mg/kg whereas CDDP was dosed daily at 1.5 mg/kg i.p. At day 15, control and treated animals were sacrificed, and the inhibition of tumor growth was evaluated. Noteworthy, administration of HydroCuP at 50 and 30 mg/kg induced 85% and 82% reduction of the tumor mass, respectively, compared to that of the control group (Table 1). A similar effect was exerted by CDDP (87% reduction), however, the time course of body weight changes indicated that CDDP induced elevated weight loss, while treatment with HydroCuP resulted in a body weight loss <10% (Fig. 2, panel B).

In the late treatment schedule with split-doses, 9 days after tumor inoculation (palpable tumor) treated animals received a higher loading dose of 50 mg/kg followed by a low maintenance dose of 30 mg/kg of HydroCuP. At this dosing regimen, the administration of HydroCuP resulted in a quite complete tumor regression (about 95%) already after the sixth administration. The results summarized in Table 1 clearly show that a complete disappearance of primary tumors in mice was confirmed by dissection on day 15. CDDP dosed at 1.5 mg/kg induced a tumor regression of 73%. The time course of body weight changes depicted in Fig. 2, panel C, indicates that treatment with HydroCuP did not induce any adverse effects including significant body weight loss throughout the therapeutic experiment.

### Tissue distribution in LLC-bearing mice

**Figure 3:**
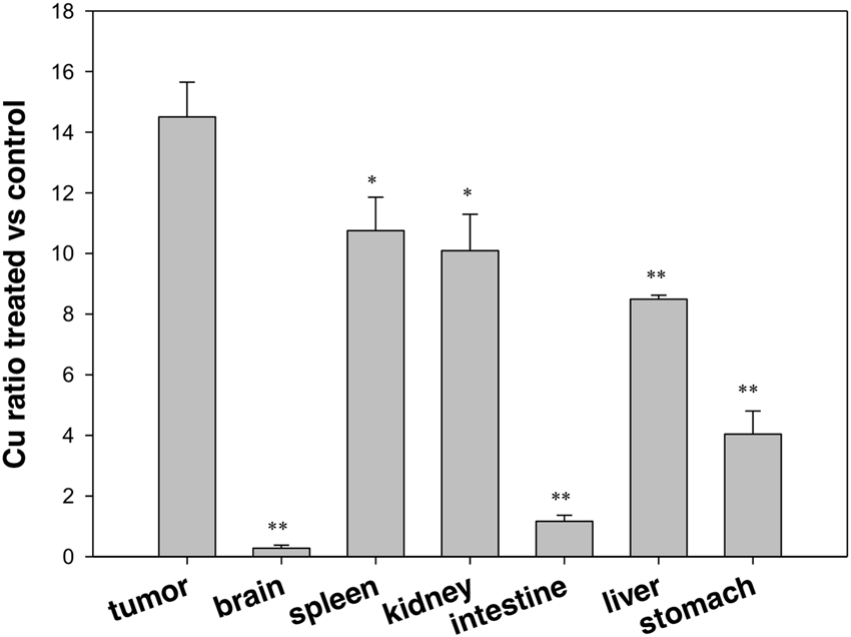
Tissue distribution in LLC bearing C57BL mice. C57BL mice were inoculated i.m. on the right flank with LLC cells (2 × 10^6^). After 10 days, HydroCuP was administered i.p. at a dose of 50 mg/kg. The mice were sacrificed after 24 hours and tumor, brain, spleen, kidney, intestine, liver and stomach were excised. Tissues were washed in ice-cold saline and weighed after removing excess fluid. All samples were mineralized in HNO_3_ and Cu content in each sample was measured by GF-AAS. Error bars indicate the S.D. **P < 0.01; *P < 0.05 (tumor vs organs).

To better understand the pharmacology and toxicology of the novel copper(I) complex, tissue samples, collected from LCC-bearing C57BL mice were analysed. Figure 3 shows tissue distribution of HydroCuP in LLC-bearing mice. On day 10 after tumor implantation, mice were treated with a single i.p. injection of 50 mg/kg of HydroCuP. After 24 hours, animals were sacrificed and copper content was quantified by graphite furnace atomic absorption spectroscopy (GF-AAS) analysis in several organs and normalized to control mice tissues. The solid tumor mass presented the highest copper concentration (at least 1.4 times higher than that detected in spleen), followed by spleen, kidney, liver and stomach. These results clearly indicate a great bioavailability to the malignant tissue of parenteral administration of HydroCuP. Interestingly, with respect to control animals, no enhance in copper content was detectable in brain, thus suggesting that HydroCuP was not able to cross the blood–brain barrier.

### *In vivo* antitumor activity toward human colorectal cancer xenografts

**Figure 4:**
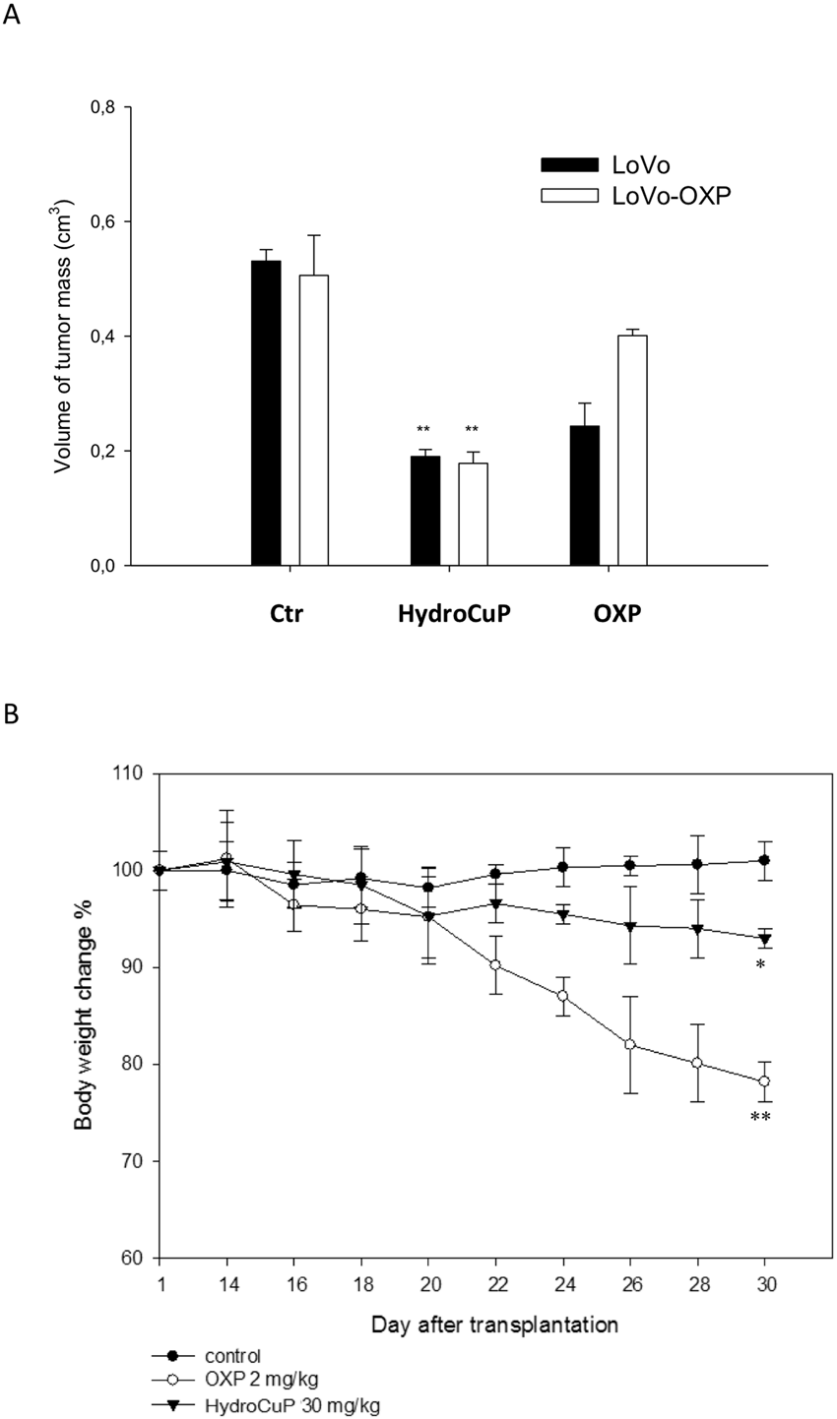
*In vivo* activity toward LoVo and LoVo-OXP tumor xenografts. (**A**) LoVo and LoVo-OXP tumor xenografts in 6-week-old BALB/c nu/nu mice by injecting 1 × 10^7^ tumor cells subcutaneously on the left dorsal flank. After 24 h from tumor implantation, mice randomly divided into six groups (6 animals per group). Chemotherapy delayed until day 14 (tumor volumes of about 0.4 cm^3^). From day 14, HydroCuP dosed daily at 30 mg/kg i.p., OXP dosed daily at 2 mg/kg i.p. At day 30, animals sacrificed, and the inhibition of tumor growth determined by comparing the volume of the control group and the treatment group expressed as percentage referred to the control animals. Error bars indicate the S.D. *p < 0.05, **p < 0.01, with respect to control. (**B**) The body weight changes of LLC-bearing C57BL mice treated with vehicle or tested compounds. Body weight was measured every two days and was taken as a parameter of systemic toxicity. The error bars indicate the S.D. **P < 0.01; *P < 0.05.

We have previously shown that HydroCuP markedly inhibited cell viability of human colon cancer cells corresponding to different stages of disease progression and mostly characterized by the predominance of antiapoptotic factors or defects in apoptosis effectors^21^. HydroCuP was also able to overcome OXP resistance in human colon cancer cells^21^. On this basis, we investigated whether these properties translated into tumor growth inhibition in preclinical *in vivo* models of human colorectal cancers. To this aim, we developed the OXP-sensitive and OXP-resistant LoVo xenografts in BALB/c nu/nu mice. OXP-resistant Lovo cells were obtained following 22 months of selection and were about 17-fold resistant to OXP^18^. OXP-sensitive or OXP-resistant LoVo cells (1 × 10^7^) were injected in the flank of mice. Once tumor was established (tumor volumes of about 0.4 cm^3^, around 14 days after tumor inoculation), mice were randomly divided into six groups (6 animals per group, 8 controls). The HydroCuP-treated groups (6 mice each group) received daily 30 mg/kg by i.p. injection and the OXP-treated group was given 2 mg/kg by i.p. injection. At day 30, animals were sacrificed, and the inhibition of tumor growth was determined (Fig. 4, panel A). Measurements of body weights were recorded from day 14 every 2 days until the experimental endpoint (Fig. 4, panel B).

Compared with tumor growth in control mice receiving vehicle (0.9% NaCl), OXP markedly delayed tumor growth (about 55%) only in LoVo model whereas in LoVo-resistant model the tumor growth inhibition reached only 20%. Conversely, HydroCuP demonstrated a statistically significant decrease in final tumor volume (about 65%) compared with the control, both in LoVo and in LoVo-resistant models (Fig. 4, panel A). No significant difference in body weight and appearance of deterioration in health was observed between the HydroCuP treated and the control group thus indicating a minimal animal toxicity (Fig. 4, panel B). In contrast, in both LoVo and LoVo-resistant xenografts, treatment with OXP induced a body weight loss of about 23%. Overall, these data confirming the HydroCuP ability to overcome OXP resistance also *in vivo* are very promising preclinical data that could suggest a new therapeutic approach against this subset of colon cancer.

### Neurotoxicity studies

As widely described in the literature, peripheral neuropathy is the most common dose-limiting toxicity of CDDP and OXP, and it is one of the major causes of discontinuation of therapy^25,26^. In particular, OXP is reported by the Food and Drug Administration to be responsible for more than 70% rate of symptomatic neurotoxicity with any severity^27^, and often leads to treatment suspension^32,33^.

**Figure 5:**
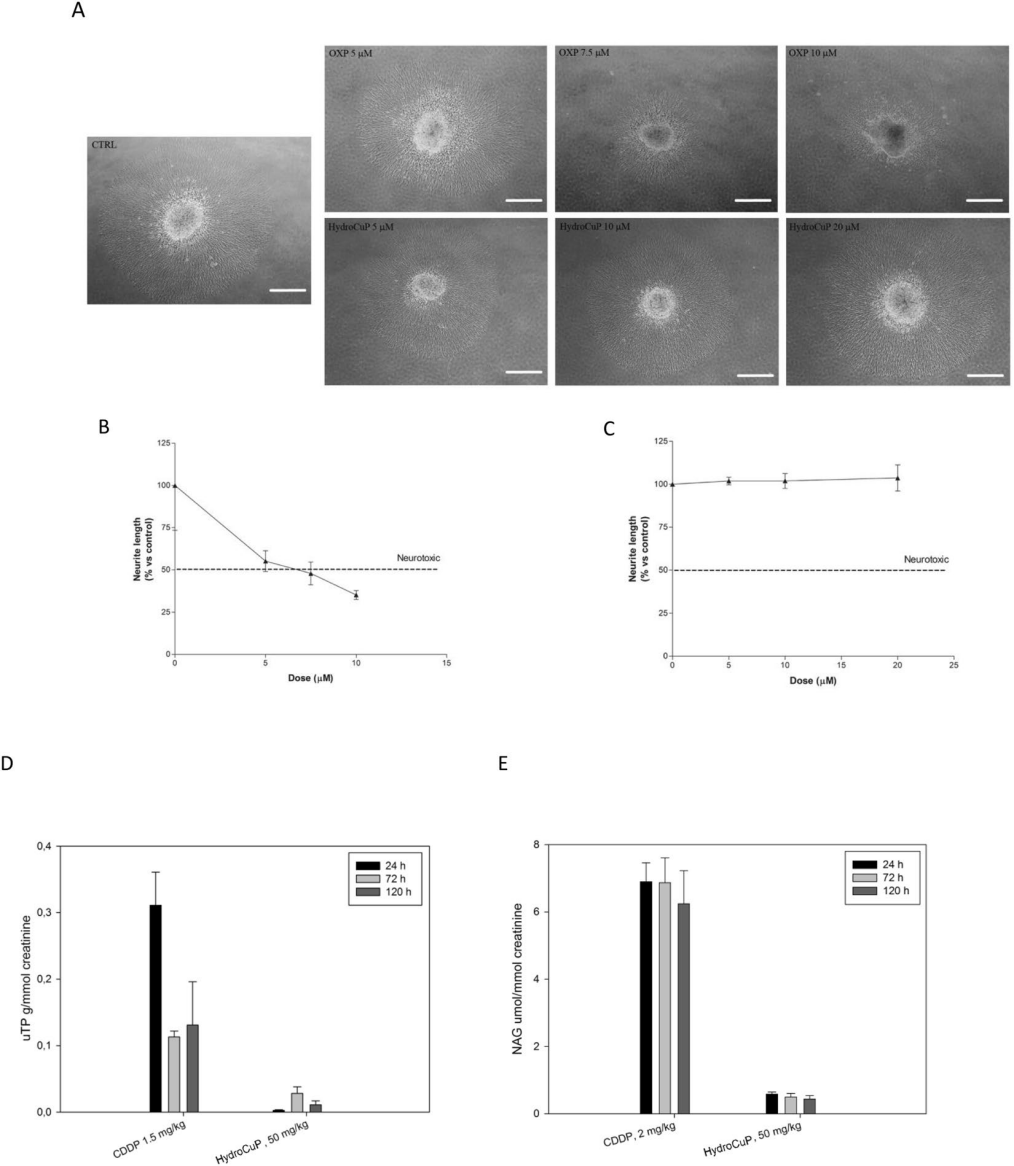
Neurotoxicity studies. OXP and HydroCuP toxicity at 48 h in DRG explants from 15 day-embryonic Sprague-Dawley rats. Representative images of neurite length in maturing DRG explants with different concentrations of OXP and HydroCuP (**A**). Bar: 500 µm. Percentage reduction curve of DRG treated with OXP (**B**) and HydroCuP (**C**) with respect to the controls (mean ± S.D., P < 0.05). Nephrotoxicity studies. Eight-week-old male Sprague Dawley rats were treated with a single i.p. injection of HydroCuP, CDDP or the vehicle solution (saline solution, control). Rats were then placed into metabolic cages and urines collected after 24, 72 and 120 h. Afterwards urines were centrifuged and aliquoted to measure creatinine, uTP (**D**) and NAG (**E**). Error bars indicate the S.D.

To investigate the potential neurotoxic effect of HydroCuP, we employed a well-established *in vitro* model based on organotypic cultures of DRG from 15-day-old rat embryos. Neurite elongation under NGF influence was evaluated in DRG explants. DRG are the neuronal structures most severely affected in platinum-induced peripheral neurotoxicity and DRG explants model is particularly useful since it permits to make prediction of potential neurotoxic effects of the tested drug in a clinical setting. This model has been widely used to study the neurotoxic effect of several anticancer drugs showing reliable results^34–37^. For comparison purposes, the effect induced by the reference drugs OXP and CDDP was also tested. As expected, after 48 h CDDP and OXP treatment significantly reduced neurite elongation in a dose-dependent manner (Fig. 5, panel A). In particular, OXP at 7.5 µM reduced by 50% neurite length (Fig. 5, panel B). On the contrary, HydroCuP showed no neurotoxic effect even at the highest concentrations (Fig. 5, panel C). These findings propose HydroCuP as a much safer agent, in terms of neurotoxicity, compared with platinum drugs.

### Nephrotoxicity studies

As stated before, for platinum compounds the irreversible and acute kidney damage is one of the main problems that occurs during clinical applications. Despite of preventive precautions, indeed, irreversible renal damage occurs in about one-third of patients under cisplatin treatment^34^.

The potential nephrotoxic effect of HydroCuP was evaluated by measuring some specific biomarkers in urines obtained from 8-week-old male Sprague Dawley rats treated with a single i.p. injection of HydroCuP and CDDP. Urines of the treated animals were collected after 24, 72 and 120 h, and urinary total protein (uTP) and N-acetyl-β-D-glucosaminidase (NAG) were evaluated as signs of nephrotoxicity. As expected, CDDP induced a significant increase of uTP excretion (Fig. 5, panel D) and NAG (Fig. 5, panel E). On the contrary, treatment with HydroCuP determined a 24 h excretion of uTP roughly 12 times lower than that recorded with CDDP (Fig. 5, panel D). Following 120 h, the levels of uTP excreted by HydroCuP were 11 times lower compared with the reference metallodrug.

On the other hand, NAG activity detected after injection with HydroCuP was up to 12 times lower compared to those detected after injection of CDDP (Fig. 5, panel E). These results clearly suggest for HydroCuP a scarce nephrotoxic potential compared to that showed by the clinically approved metallodrug, CDDP.

### Histopathological Examination

We previously demonstrated that HydroCuP is able to induce paraptosis in human LoVo colon cancer cells by triggering ER stress leading to the UPR^21^. In particular, treatment with HydroCuP *in vitro* was found to provoke a time-dependent increase in the phosphorylation of both protein kinase R-like endoplasmic reticulum kinase (PERK) and inositol requiring enzyme (IRE-1), two of the major transducers of endoplasmic reticulum (ER) stress^21^.

In the initial phases of UPR induction, the activated cytosolic domain of PERK phosphorylates the eIF2alpha, inhibiting nuclear translation and resulting in cell death. In addition, the activated cytosolic domain of IRE1 acts on its substrate XBP1 which translocating into the nucleus activating UPR-target genes^21^.

**Figure 6:**
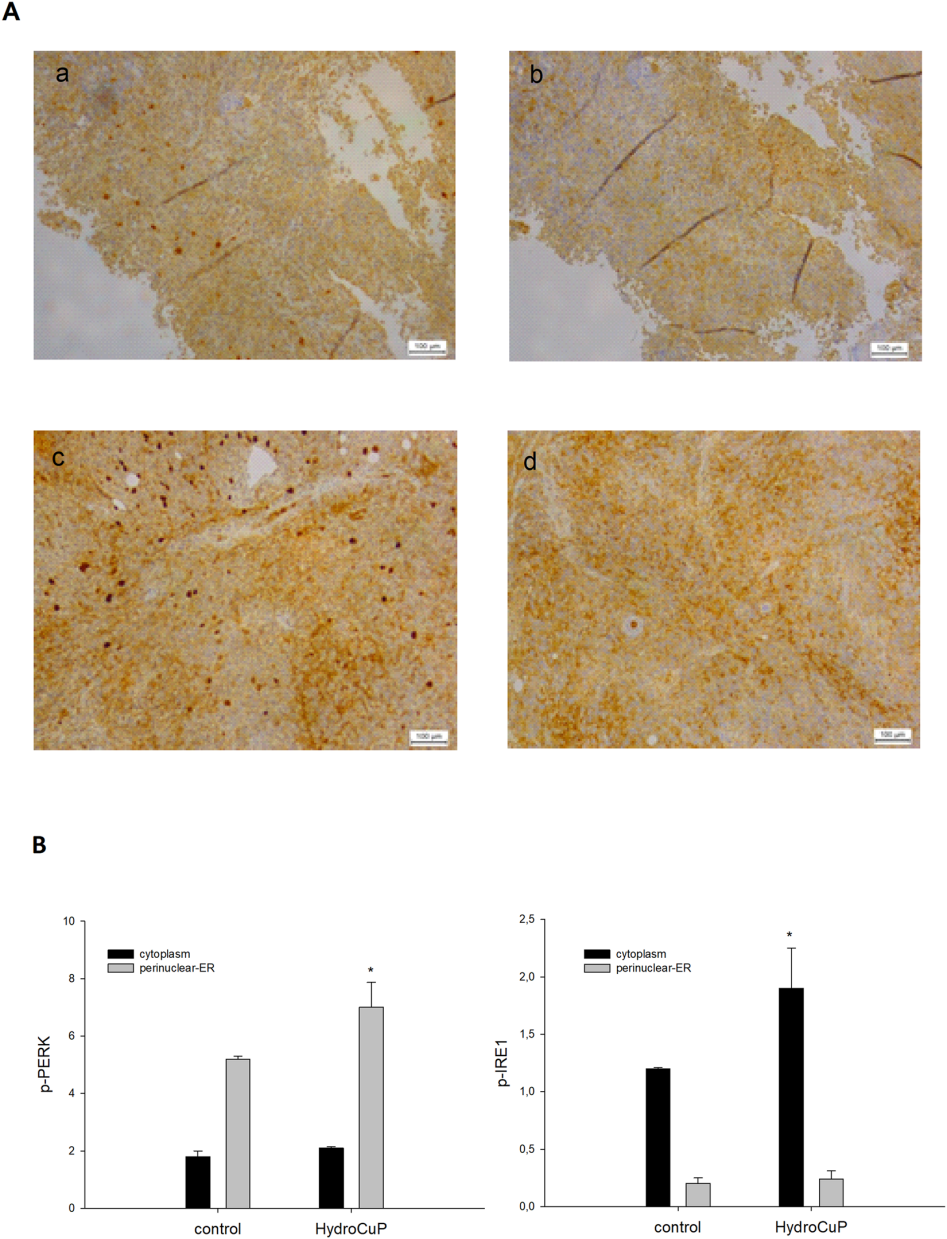
IHC analysis of p-PERK and p-IRE1 expression in LLC tumor samples. (**A**) Pictures show representative patterns of p-PERK and p-IRE1 expression by IHC in control LLC tumor samples (a and c) and in HydroCuP-treated tumor samples (b and d); magnification: ×10. (**B**) p-PERK (a) and p-IRE1 (b) values, evaluated in LLC tumor sections. The quantification of IHC images was performed by using Image J software. Columns indicate quantitative analysis in n = 3 different tumors per group; *P < 0.05.

In order to investigate the *in vivo* activation of the UPR induced by HydroCuP, the levels of the phospho-PERK (p-PERK) and phospho-IRE1 (p-IRE1) were detected in tumor samples derived from treated LCC-bearing mice (following later-stage regimen). Figure 6 shows microphotographs of control and HydroCuP-treated tumor samples by IHC analysis. In control samples, p-PERK staining was found moderate in cytoplasm (in about 40% cell population) and intense in perinuclear-ER (about 5% of cell population). IHC of tumor exposed to HydroCuP treatment revealed no difference in cytoplasmic p-PERK positivity whereas perinuclear-ER p-PERK staining significantly increase (about 30%) in comparison to controls. With regard to the downstream p-PERK effector, p-IRE1, control tumors were weakly positive in cytoplasm (in about 15% cell population). HydroCuP-treated tumor samples showed a marked increase in cytoplasmic positivity (about 35% cell population) in p-IRE1 IHC. Overall, the intense perinuclear-ER p-PERK immunoreactivity and the substantial increase of cytoplasmatic p-IRE1 observed in tumor sections suggest the involvement of this effectors in the cell death mechanism induced by HydroCuP.

These IHC data are consistent with our previous *in vitro* observations, and confirm that HydroCuP acts *in vivo* by inducing the UPR pathway.

## Conclusions

The systematic studies of medicinal chemistry aimed at identifying new metallodrugs led us to select HydroCuP, a phosphino copper(I) complex highly soluble and stable in physiological media. HydroCuP has exquisite potency against human cancer cells derived from solid tumors, being preferably effective towards malignant rather than non-malignant cells. In agreement with our previous *in vitro* data, here we confirmed by using 3D-cultures that HydroCuP strongly inhibited cell growth of human colon cancer cells. More importantly, HydroCuP inhibited tumor growth in preclinical models much strongly with respect to both platinum reference drugs. Tested against the highly aggressive, poorly immunogenic murine LLC, HydroCuP caused a quite complete tumor regression, and towards human colorectal cancer xenografts, chemotherapy with HydroCuP was highly effective in both OXP-sensitive and -resistant models, thus attesting HydroCuP ability to overcome OXP-resistance. To the best of our knowledge, this is the first example of a copper(I) complex reported to inhibit cell growth of OXP-resistant cancer cells in an *in vivo* tumor model. The histopathological results, confirming that HydroCuP acted by inducing the UPR pathway, were in line with those already obtained in cultured LoVo cells. In addition, contrary to platinum drugs, treatment with HydroCuP did not induce severe adverse effects including significant body weight loss. The favorable *in vivo* tolerability of HydroCuP was correlated to an encouraging biodistribution profile demonstrating that it accumulated preferentially in the tumor mass. Experiments on DRG organotypic cultures treated with HydroCuP did not show evidence of peripheral neurotoxicity whereas the monitoring of urinary biomarkers for nephrotoxicity did not track renal injury in HydroCuP treated mice. Even though further organ toxicity assessments are needed, these latter preclinical data are very promising, as neurotoxicity and nephrotoxicity are the most severe dose-limiting side effects associated with platinum drug chemotherapy. Altogether, our results demonstrate that HydroCuP appears worth of further investigation to evaluate its therapeutic activity towards a broad spectrum of solid malignancies.

## Methods

### Experiments with human cells

HydroCuP, CDDP and OXP (Sigma Chemical Co.) were dissolved in 0.9% NaCl solution just before the experiment.

### Cell line and spheroid culturing

Human colon (HCT-15 and LoVo) carcinoma cell lines were obtained by American Type Culture Collection (ATCC, RocKville, MD). Cells were routinely cultured in RPMI-1640 medium (Sigma Chemical Co.) containing 10% foetal heat-inactivated calf serum (FCS; Euroclone, Milan, Italy), antibiotics (50 units/mL penicillin and 50 mg/mL streptomycin) and 2 mM L-glutamine. All cultures were kept at 37 °C in a humidified atmosphere with 5% CO_2_. Cell transfer and preparation of single-cell suspensions were performed by mild enzymatic dissociation using a 0.05% trypsin and 0.02% EDTA solution in PBS (Euroclone, Milan, Italy).

Spheroids were initiated in liquid overlay by seeding 2.5 × 10^3^ HCT-15 and LoVo cells/well in phenol red-free RPMI-1640 medium (Sigma Chemical Co.), containing 10% FCS and supplemented with 20% methyl cellulose stock solution. A total of 150 μl of this cell suspension was transferred to each well of a round bottom non-tissue culture treated 96 well-plate (Greiner Bio-one, Kremsmünster, Austria) to allow spheroid formation within 72 h.

### Cell viability assays

A modified APH assay, which is based on quantification of cytosolic acid phosphatase activity, was used for determining cell viability in spheroids^38^. Briefly, the pre-seeded spheroids were treated with fresh medium containing the compound to be studied at the appropriate concentration (range 5–150 μM). Triplicate cultures were established for each treatment. After 72 h, each well was treated with 100 μL of the assay buffer (0.1 M sodium acetate, 0.1% Triton-X-100, supplemented with ImmunoPure p-nitrophenyl phosphate; Sigma Chemical Co.) and, following 3 h of incubation, 10 μL of 1 M NaOH solution were added. The inhibition of the cell growth induced by the tested complexes was detected by measuring the absorbance of each well at 405 nm, using a Bio-Rad 680 microplate reader. Mean absorbance for each drug dose was expressed as a percentage of the control untreated well absorbance (T/C) and plotted vs drug concentration. IC_50_ values, the drug concentrations that reduce the mean absorbance at 405 nm 50% of those in the untreated control wells, were calculated by four-parameter logistic (4-PL) model. Evaluation was based on means from at least four independent experiments.

### *In vivo* experiments

All experiments were performed according to D.L.vo 26/2014, which regulates the use of experimental animals in Italy. The research project was approved by the Italian Health Department according to the art. 20 of above mentioned D.L.vo.

#### *In vivo* anticancer activity toward LLC

The mice were purchased from Charles River, Italy, housed in steel cages under controlled environmental conditions (constant temperature, humidity, and 12 h dark/light cycle), and alimented with commercial standard feed and tap water ad libitum. The LLC cell line was purchased from ECACC, United Kingdom. The LLC cell line was maintained in DMEM (Euroclone, Pero, Italy) supplemented with 10% heat-inactivated foetal bovine serum (Euroclone, Pero, Italy), 10 mM L-glutamine, 100 U/mL penicillin, and 100 µg/mL streptomycin in a 5% CO_2_ air incubator at 37 °C. The LLC was implanted intramuscularly (i.m.) as a 2 × 10^6^ cell inoculum into the right hind leg of 8-week old male and female C57BL mice (24 ± 3 g body weight). The antitumor activity of HydroCuP in LLC tumor model has been investigated by means of different schedules (early treatment, intermediate treatment and late treatment) and compared with that promoted by the reference metallodrug, CDDP. In the early-stage treatment, animals were treated at day 3, 5, 7, 9, 11 and 13 after the tumor cell inoculum with an i.p. injection of HydroCuP (25, 35 and 50 mg/kg), CDDP (1.5 mg/kg) or the vehicle solution (0.9% NaCl).

In the intermediate treatment, animals were treated after 7 days from tumor implantation (visible tumor) with daily i.p. doses of HydroCuP (30 and 50 mg/kg), CDDP (1.5 mg/kg) or the vehicle solution (0.9% NaCl). In the late treatment, animals were treated after 9 days from tumor implantation (palpable tumor) with i.p. doses of HydroCuP (50 mg/kg) from day 9 to day 11 and with i.p. doses of HydroCuP (30 mg/kg) from day 12 to day 14, or with daily doses of CDDP (1.5 mg/kg) or with the vehicle solution (0.9% NaCl). At day 15, animals were sacrificed, the legs were amputated at the proximal end of the femur, and the inhibition of tumor growth was determined according to the difference in weight of the tumor-bearing leg and the healthy leg of the animals expressed as a percentage referring to the control animals. Body weight was measured every 2 days and was taken as a parameter for systemic toxicity.

#### Biodistribution studies in LLC-bearing mice

C57BL mice were inoculated i.p. on the right flank with LLC cells (2 × 10^6^). After 10 days from tumor implantation, HydroCuP was administered i.p. at a dose of 50 mg/kg. The mice were sacrificed after 24 h and tumor, brain, spleen, kidney, intestine, liver and stomach were excised. Tissues were washed in ice-cold saline and weighed after removing excess fluid. All samples were mineralized in HNO_3_ and Cu content in each sample was measured by GF-AAS (Graphite Furnace Atomic Absorption Spectroscopy).

#### *In vivo* anticancer activity toward colorectal oxaliplatin-sensitive and –resistant xenograft models

LoVo and LoVo-OXP tumor xenografts were established in 6-week-old BALB/c nu/nu mice by injecting 1 × 10^7^ tumor cells subcutaneously (100 μL in serum free medium) on the left dorsal flank. After 24 h from tumor implantation, mice were randomly divided into six groups (6 animals per group, 8 controls). Chemotherapy was delayed until the tumor was about 0.4 cm^3^ (day 14). From day 14, HydroCuP was dosed daily at 30 mg/kg i.p. whereas OXP was dosed daily at 2 mg/kg i.p. Measurements of body weights and tumor volumes were recorded from day 14 every 2 days until the experimental endpoint. The long axis (L) and the short axis (S) were measured with callipers, and the tumor volume (V) was calculated using the following equation: V = SxSxL/2. At day 30, animals were sacrificed, and the inhibition of tumor growth was determined by comparing the volume of the control group and the treatment group expressed as percentage referred to the control animals.

#### Nephrotoxicity studies

Eight-week-old male Sprague Dawley rats were randomly allocated to five groups (five animals per group) and treated with a single i.p. injection of HydroCuP (50 mg/kg) or the vehicle solution (0.2 mL saline solution, control). Cisplatin (1.5 mg/kg,) was also used under the same experimental conditions for comparison purposes. Rats were then placed into metabolic cages and urines collected after 24, 72 and 120 h. Later, urines were centrifuged (150 *g* for 10 minutes at room temperature) to discard debris and aliquoted to measure creatinine, uTP and NAG. Urine creatinine assays were performed using creatinine assay kit from Sigma Chemical Co. (St. Louis, MO). uTPs were measured by means of BioRad Total Protein Test (Hercules, CA). Urinary NAG was measured spectrophotometrically with the NAG kit (Roche diagnostics, Basel, Switzerland) according to the manufacturer’s protocols. uTP and NAG are expressed as grams per millimoles of creatinine (g mmol^−1^ creatinine).

#### DRG Explants and Assessment of Neurite Outgrowth from DRG

DRG from E15 Sprague-Dawley rats (Envigo, San Pietro al Natisone, Italy) were aseptically removed and cultured onto a single layer of rat tail collagen surfaces in 35-mm dishes as previously described^36^.

The DRG were incubated in AN2 medium [MEM added with 1.4 mM L-glutamine (Euroclone, Pero, Italy), 10% calf bovine serum (Hyclone, Thermo Scientific, Logan, UT) 50 µg/ml ascorbic acid, 0.6% glucose (Sigma-Aldrich) in the presence of 5 ng/ml nerve growth factor (NGF; Life Technologies, Monza, Italy) in a 5% CO_2_ humidified incubator at 37 °C.

To evaluate the neurotoxicity of HydroCuP and OXP, the DRG explants were treated for 2 h with NGF and subsequently exposed to each drug at different concentrations for 48 h. OXP was tested at 5, 7.5 and 10 μM while 5, 10 and 20 μM of HydroCuP was used. DRG treated with AN2 medium supplemented with 5 ng/ml NGF alone were used as controls. Phase-contrast micrographs were taken, and the length of the longest neurite in each DRG was measured by Image J (NIH, Bethesda, MD), using a standard calibration grating photographed at the same magnification. A compound is considered neurotoxic when the mean neurite elongation is reduced by 50% or more after drug exposure vs control.

### Immunohistochemical Analysis

To evaluate p-PERK and p-IRE1 expression, five-micron-thick formalin-fixed, paraffin-embedded (FFPE) tumor samples of LLC models (3 tumors/group, 3 sections/tumor and 3 images/sections) were stained by IHC using anti-human p-PERK (Thr981, Santa Cruz) or p-IRE1 (Ser724, Abcam) primary antibodies according to the manufacturer’s instructions. IHC was performed using a Leica Bond III Autostainer (Leica). Antigen retrieval was performed in citrate buffer for 15 min. Sections were counter-stained with Mayer’s haematoxylin. IHC results were evaluated by one experienced pathologist with no prior knowledge of experimental data. The quantification of IHC images was performed by using Image J software.

### Statistical analysis

All values are the means ± SD of no less than three measurements starting from three different cell cultures. Multiple comparisons were made by ANOVA followed by the Tukey−Kramer multiple comparison test (**P < 0.01; *P < 0.05), using GraphPad Software.
